# CTX-M Extended-spectrum β-Lactamases, Washington State

**DOI:** 10.3201/eid1303.060479

**Published:** 2007-03

**Authors:** Seonghan Kim, Jinxin Hu, Romesh Gautom, Junyoung Kim, Bokkwon Lee, David S. Boyle

**Affiliations:** * Washington Department of Health, Shoreline, Washington, USA; † Korea Centers for Disease Control and Prevention, Seoul, Republic of Korea

**Keywords:** Extended-spectrum β-lactamases, CTX-M, Washington, letter

**To the Editor:** The CTX-M–type β-lactamases are non-TEM and non-SHV plasmid-mediated, class A, extended-spectrum β-lactamases (ESBLs). The CTX-M–type β-lactamases have recently emerged as the most common type of ESBLs, with a global distribution (*1*). In contrast, the CTX-M–type ESBLs are rarely reported in the United States and have not been identified in pathogens isolated from infected patients with gastroenteritis.

We screened 637 *Salmonella* and 126 *Shigella* isolates, collected in the state of Washington during 2003–2004, for CTX-M–type β-lactamases. Of these, 60 *Salmonella* isolates that exhibited an ESBL phenotype were further characterized by PCR for TEV, SHV, CTM-X, and CMY. All were positive for the CMY-2 or TEM-1 β-lactam genes. One *Shigella sonnei* isolate (WA7593), cultured from a fecal specimen in August 2004, tested positive with an ESBL confirmatory disk diffusion panel (ceftazidime 24 mm, ceftazidime/clavulanate 32 mm, cefotaxime 14 mm, and cefotaxime/clavulanate 34 mm; [*2*]). The patient had recently traveled to Pakistan and likely became ill there and returned to the United States while still sick. The transfer of extended-spectrum cephalosporin resistance was tested by conjugation to *Escherichia coli* J53 azi^R^ (*3*). The MIC for *S. sonnei* WA7593 and its transconjugant, WA7593TC1, were tested by using the E-test (AB Biodisk, Solna, Sweden). Both strains were resistant to cefotaxime and susceptible to ceftazidime and showed almost the same antimicrobial susceptibility patterns as β-lactam antimicrobial drugs (Table).

**Table:** MICs of antimicrobial drugs for *Shigella sonnei* clinical isolate WA7593 and its transconjugant WA7593TC1

Antimicrobial drug	MIC (μg/mL)	
	WA7593	WA7593TC1
Ampicillin	>256	>256
Cephalothin	>256	>256
Cefotaxime	>32	>32
Ceftazidime	4	4
Ceftriaxone	>32	>32
Cefaclor	>256	>256
Imipenem	0.19	0.25
Trimethoprim/sulfamethoxazole	>32	0.032

The type of ESBL produced by these strains was determined by using PCR specific for TEM and CTX-M (*4*,*5*). Both strains were PCR positive for TEM and CTX-M. The TEM type PCR products were then sequenced and identified as TEM-1; no variation was found on the promoter region of *bla*_TEM-1_. The entire sequence of *bla*_CTX-M_ from WA7593 was then sequenced (*1*), and the product showed 100% homology with *bla*_CTX-M-15_ (GenBank accession no. AY960984). The mobile element associated with the transfer of *bla*_CTX-M-15_ was investigated by sequencing the flanking regions. PCRs were performed with primers from the internal regions of *bla*_CTX-M_ gene and primers for insertion sequences IS*Ecp1* and IS*903* (*4*,*5*). Positive PCR products were obtained with primers IS*Ecp1*F and CTX2 (943 bp); no amplified product was produced with primers CTX1 and IS*903*R. Sequencing of a 943-bp amplicon showed that *bla*_CTX-M15_ was flanked upstream by an IS*Ecp1-*like element.

The presence of an integron in *S. sonnei* WA7593 and WA7593TC1 was investigated by using integron-specific primers hep35 and hep36 (*2*). Only *S. sonnei* WA7593 produced a PCR product. This finding suggests that the transmission of *bla*_CTX-M15_ is not by integron-mediated transfer. A further 162 *Shigella* spp. and 260 *Salmonella* spp. isolated from 2003 through 2005 were also screened for ESBL production; no further isolates were identified.

The presence of a CTX-M–type, ESBL-producing isolate is rarely reported in the United States. The only other reference was from a multistate study in 2001–2002 that identified CTX-M type from *E. coli* isolates from urine, sputum, and blood (*6*). No further reports about CTX-M–producing organisms have been disseminated. Our investigation suggests that CTX-M–type ESBLs may spread throughout the United States through infected travelers. This finding is notable because *S. sonne*i is a common enteric pathogen. Our results further emphasize that travelers from others parts of the world can introduce highly mobile and clinically important resistance mechanisms into the community. The spread of CTX-M ESBLs may be faster and more widespread than previously thought; therefore, CTX-M type should be taken seriously as a surveillance target in the United States, especially in patients with a history of travel outside North America.
